# Recent Advancements in the Application of Circulating Tumor DNA as Biomarkers for Early Detection of Cancers

**DOI:** 10.1021/acsbiomaterials.4c00606

**Published:** 2024-07-01

**Authors:** Mahima Mishra, Rubai Ahmed, Deepak Kumar Das, Devlina Das Pramanik, Sandeep Kumar Dash, Arindam Pramanik

**Affiliations:** † Amity Institute of Biotechnology, Amity University, Noida 201301, India; ‡ Department of Physiology, University of Gour Banga, Malda-732103, West Bengal, India; § Department of Chemistry and Nanoscience, GLA University, Mathura, 281406 Uttar Pradesh, India; ∥ School of Medicine, University of Leeds, Leeds LS53RL, United Kingdom

**Keywords:** Circulating tumor DNA, Cell free DNA, Cancer Biomarker, DNA methylation, DNA probes, RNA probes

## Abstract

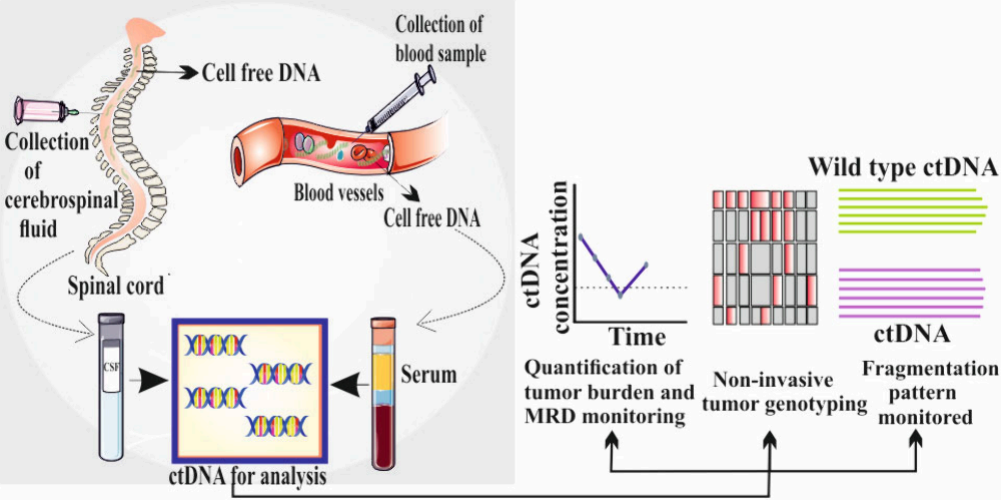

Early detection of cancer is vital for increasing patient survivability chances. The three major techniques used to diagnose cancers are instrumental examination, tissue biopsy, and tumor biomarker detection. Circulating tumor DNA (ctDNA) has gained much attention in recent years due to advantages over traditional technology, such as high sensitivity, high specificity, and noninvasive nature. Through the mechanism of apoptosis, necrosis, and circulating exosome release in tumor cells, ctDNA can spread throughout the circulatory system and carry modifications such as methylations, mutations, gene rearrangements, and microsatellite instability. Traditional gene-detection technology struggles to achieve real-time, low-cost, and portable ctDNA measurement, whereas electrochemical biosensors offer low cost, high specificity alongside sensitivity, and portability for the detection of ctDNA. Therefore, this review focuses on describing the recent advancements in ctDNA biomarkers for various cancer types and biosensor developments for real-time, noninvasive, and rapid ctDNA detection. Further in the review, ctDNA sensors are also discussed in regards to their selections of probes for receptors based on the electrode surface recognition elements.

## 1 Introduction

Early cancer detection is critical for increasing survival and treatment outcomes. When cancer is detected at an early stage, it is often more localized and less likely to have spread to other parts of the body, making treatment more successful.^1^ This early intervention may significantly improve the success rate of treatments like as surgery, chemotherapy, and radiation therapy, while also minimizing the severity and duration of adverse effects.^1^ Furthermore, early detection of cancer can reduce total healthcare costs and enhance patients’ quality of life by allowing for less aggressive therapy and minimizing the psychological and physical burden of advanced disease.^2^

Although early cancer diagnosis and detection can be difficult, it is now possible due to advancement in molecular biomarkers. To identify molecular signatures such as genetic and epigenetic changes in gene expression as well as protein expression, numerous genomic and proteomic techniques are being used. Such techniques include real-time PCR, DNA sequencing, microarrays, molecular tests, endoscopy,biopsy, artificial intelligence, and machine learning.^3^ Early in the course of the disease, many malignant tumors release intracellular molecules, whole cells or pieces of cells into the surrounding environment. These compounds can frequently be found in the blood, body fluids, or feces, which opens the door to simpler, less invasive screening techniques for the early detection of cancer. Liquid biopsies have recently gained popularity among researchers due to their noninvasive, rapid, and comprehensive nature.^2^ These liquid biopsies include detection of circulating tumor cells (CTCs), circulating tumor DNA (ctDNA), and circulating cell-free DNA (cfDNA).

**Figure 1:**
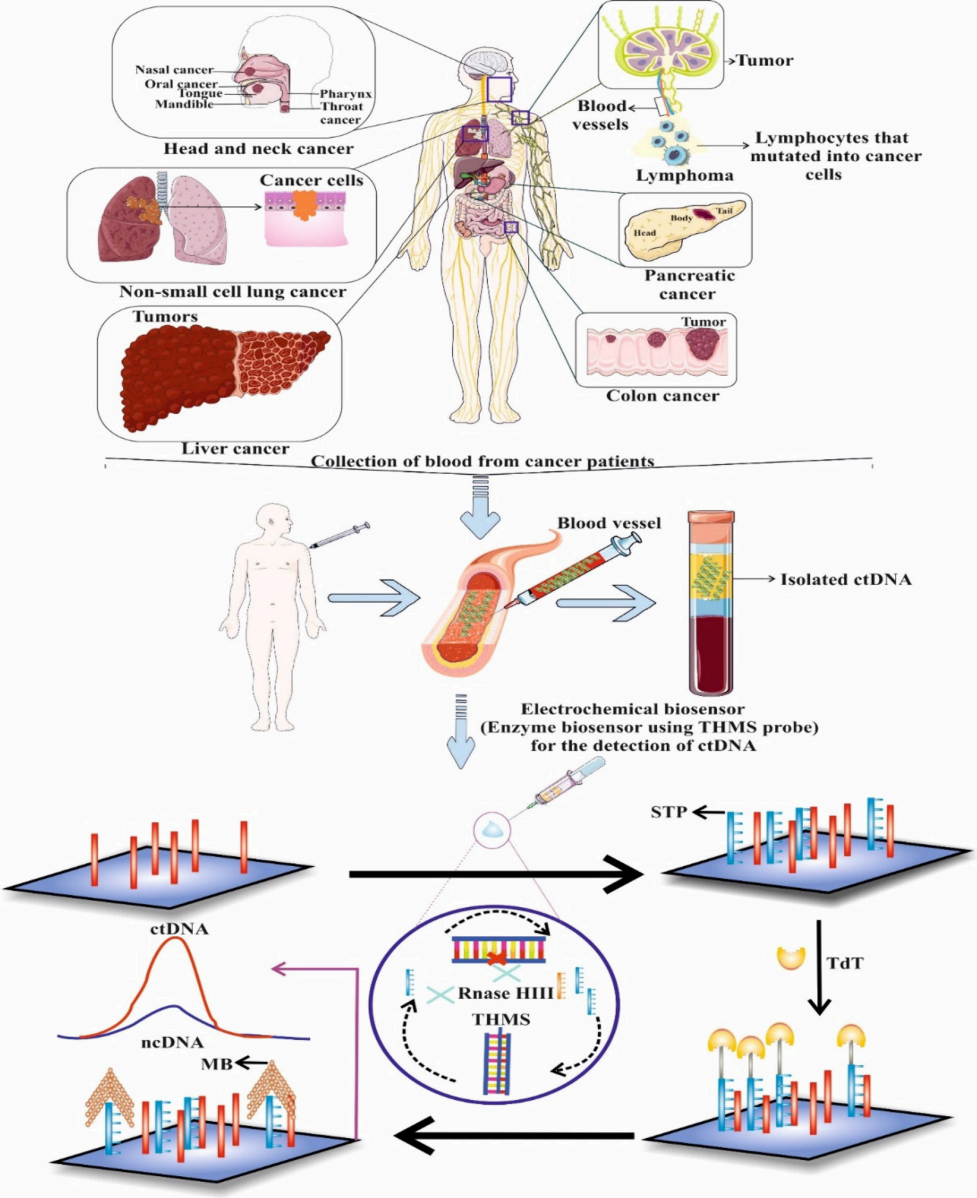
This figure represents blood samples collection from various cancer patients, isolation of ct-DNA and detection of specific cancer via electrochemical biosensing. Parts of the figure have been drawn by using pictures from Servier Medical Art. Servier Medical Art by Servier is licensed under a Creative Commons Attribution 3.0 Unported License (https://creativecommons.org/licenses/by/3.0/.

Tumor cell DNA circulating in the blood, commonly referred to as circulating tumor DNA, or ctDNA, is one kind of biomarker that has attracted much attention. Because of its distinct features and benefits, ctDNA testing stands out among early cancer detection approaches.^4^ Unlike typical diagnostic tests, ctDNA testing is a noninvasive way to detect tumor-derived genetic material in the circulation. This groundbreaking approach gives clinicians crucial insights into tumor features and behavior with unparalleled precision. Clinicians can use ctDNA analysis to track disease development, predict therapy response, and detect minor residual illness or relapse earlier than traditional approaches.^5^ This noninvasive liquid biopsy could be performed using plasma/serum, uterine lavage, and urine samples. Furthermore, ctDNA testing allows for personalized treatment plans, assisting doctors in selecting targeted medicines and more efficiently monitoring treatment efficacy.^4^ In the field of early tumor diagnosis, ctDNA testing is emerging as a transformational tool, offering better outcomes and more personalized therapy **(**Figure 1**)**.

## 2 Utilizing the ctDNA in Cancer Diagnosis and Biomarkers in Various Cancers

### 2.1 Head and Neck Squamous Cell Carcinoma

Head and neck squamous cell carcinoma (HNSCC), a cancer that develops in the upper aerodigestive tract and affects the cells lining the surface of the head and neck. It is commonly associated with increased exposure to factors that cause DNA damage along with decreased function of DNA protection mechanisms. HNSCC encompasses a range of tumors originating from mucosal tissues. Oropharyngeal carcinomas are becoming increasingly common in young patients, and human papillomavirus infection (HPV) has been identified as a major contributor to their development. An estimated 560,000 new cases are diagnosed with HNSCC annually, and the disease is estimated to cause 300,000 deaths. This represents a significant annual burden of the disease.^6^

#### 2.1.1 Circulating Tumor DNA as a Biomarker for HNSCC

As discussed above ctDNA holds great promise for frequent disease progression monitoring as a minimally invasive liquid biopsy method. This, in turn, could offer valuable insights for determining the necessary level of clinical and radiological vigilance, enabling early identification and treatment of recurrent disease.^7,8^

**Figure 2:**
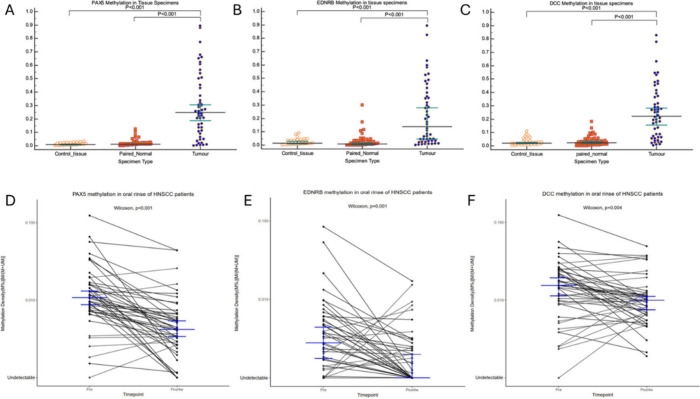
Methylation density of tumor suppressor gene markers in HNSCC tissue samples for (A)PAX5, (B) EDNRB and (C) DCC. All three markers showed aberrant methylation in tumor, compared with the paired normal tissues; Change in methylation density of tumor suppressor gene (TSG) markers in oral rinse specimens before and after surgical treatment for (D)PAX5, (E) EDNRB and (F) DCC. All markers show significant drop in the methylation densities after completion of the surgery. All three markers showed aberrant methylation in tumor, compared with the paired normal tissues (*p* < 0.001, Mann–Whitney U test) and with the control tissues (*p* < 0.001, Mann–Whitney U test). Each dot in the three groups represents individual patients. Adapted with permission from ref (12) Copyright © 2021 The Authors. Head & Neck published by Wiley Periodicals LLC..

Researchers have looked into ctDNA in blood samples related to HNSCC, but they have also looked into saliva samples.^9^ DNA from the basal (lower) and apical (upper) sides of tumor cells is released, and this DNA can be found in the blood and saliva, respectively.^10^ Wang et al. investigated the potential of ctDNA from various body sites to diagnose and monitor HNSCC.^10^ 80% of individuals with oral cavity tumors and 86–100% of patients with tumors in other areas had ctDNA found in their plasma samples.^10^ Gene methylation and other tumor-specific genetic alterations can be used to distinguish between tumor-derived ctDNA and nontumor cfDNAin HNSCC patients.^11^ In a study by Fung et al., the methylation of tumor suppressor genes in the oral rinses of HNSCC patients and controls was assessed for the application of droplet digital PCR (ddPCR) for early disease identification and monitoring.^12^ In the study, the level of methylation of the markers *PAX5(*high methylation tumor-specific marker*), Deleted in Colorectal Cancer* (DCC) and *Endothelin Receptor beta* (EDNRB) in HNSCC samples and matching pretreatment oral rinses was investigated **(**Figure 2**)**. The marker PAX5, demonstrated a specificity of 87.9% and sensitivity of 84.0% in oral rinses. The study also found that, in at least one of the evaluated markers, 76.9% of cases of relapse showed a rebound of methylation above the levels before surgery, preceding verified recurrence. This suggests that monitoring the methylation levels of these indicators in oral rinses may be an effective way to predict recurrence and provide guidance for therapy in people with HNSCC.^12^ ddPCR method was used to analyze the PAX5 methylation in deep surgical margin samples from 82 individuals with HNSCC that were histologically cancer-free.^13,14^

In a study, Galot et al. looked into the viability of liquid biopsy in locating ctDNA mutations that would be amenable to treatment.^14^ According to their research, targeted Next-Generation Sequencing (NGS) found that 30% of people with recurring nonmetastatic cancer and 70% of people with metastatic diseases had mutant ctDNA.^14^ The study revealed specific individuals with PIK3CA mutations as well as genetic changes in ctDNA that were not seen in the associated tumor tissue.

Concurrent with the clinical trial, a biomarker study involving ctDNA analysis revealed that tumor protein p53 (TP53) mutations and HPV-negative status were linked to increased benefit from combination therapy. This shows that suppressing phosphatidylinositol 3-kinase (PI3K), which historically has been associated with worse clinical outcomes, may improve outcomes in this particular group of individuals. Patients with a low tumor mutational burden (TMB) showed a better response to buparlisib and paclitaxel, in contrast to studies using checkpoint inhibitors, where patients with a high TMB tended to respond better to treatment.^15^ In patients with R/M (recurrent and/or metastatic) HNSCC, the use of liquid biopsies for ctDNA analysis can offer important insights into tumor evolution and resistance development, which in turn can enhance clinical management and treatment results.

#### 2.1.2 HPV ctDNA as a Biomarker

It is possible to use virus-originated ctDNA as a biomarker to determine if HNSCC patients have HPV or EBV (Epstein–Barr virus) infection. One example is the identification of circulating human papillomavirus (HPV) DNA, which has been shown to correlate with the amount or stage of tumors. This finding might be useful for monitoring the disease’s progression and offering information to guide therapy choices.^9,16^ Siravegna et al., investigated the efficacy of utilizing ctDNA detection as a noninvasive and low cost detection method for HNSCC patients with HPV. 61 patients with an untreated HNSCC diagnosis, and 70 HPV-negative controls were included in the study.^17^ In the initial diagnostic attempt, the success rate for diagnosis stood at 72%. When it came to diagnosing HPV-positive HNSCC, serum HPV ctDNA detection exhibited an impressive specificity of 98.6% and sensitivity of 98.4%. HPV ctDNA showed better detection accuracy when compared to the overall performance of the standard clinical assessment during the initial diagnostic attempt.^17^

Research has highlighted the effectiveness of ctDNA in identifying human papillomavirus (HPV) ctDNA within the bloodstream, exhibiting a high level of accuracy in diagnosing HPV-positive cancer cases. Moreover, the identification of HPV ctDNA in plasma has been observed to take place multiple years before the formal clinical diagnosis of HPV-positive oropharyngeal squamous cell carcinoma (OPSCC), indicating its potential as an early indicator of precursor lesions. Importantly, studies have reported a notably high degree of specificity in the detection of HPV ctDNA in plasma, with certain studies showing no instances of false positives. Numerous studies indicate that HPV ctDNA may be used as a biomarker to help patients select the best course of action.^9^

### 2.2 Pancreatic Cancer

With a limited chance of survival, pancreatic cancer (PC) is a very aggressive disease. It mostly develops from the pancreatic ductal epithelium and can spread to the liver, peritoneum, lungs, and skin, among other organs.^18^ The most prevalent type of pancreatic cancer, pancreatic ductal adenocarcinoma, requires surgical excision with tumor-free margins for the best possible treatment.^19^ The Global Burden of Disease Study 2017, vividly illustrated the global burden of PC, which is a rapidly rising cases of cancer mortality.^20^ From 1990 to 2017, the incidence of pancreatic cancer more than doubled, mostly as a result of population aging. Age is the biggest risk factor for pancreatic cancer. The global burden of PC is anticipated to keep growing as people live longer.^21^ 4.5% of cancer fatalities globally in 2018 were due to pancreatic cancer.^22^

#### 2.2.1 ctDNA as a Biomarker for Pancreatic Cancer

Tissue biomarkers in PC might be replaced by blood biomarkers. CA19–9 is the sole serum biomarker authorized by worldwide standards to track PC response.^23^ PC is characterized by genetic and epigenetic changes specific to tumors, including common mutations in the four driver genes: TP53, KRAS, CDKN2A and SMAD4. Due to their presence in ctDNA in the blood, these mutations may function as prognostic and predictive biomarkers in both early and advanced disease.^24,25^

Genetic changes known as KRAS mutations are frequently observed in pancreatic cancer cells and are linked to poor prognosis and treatment resistance.^26^ KRAS mutations in ctDNA have been identified as a promising novel biomarker for advanced pancreatic cancer, enabling noninvasive surveillance of the disease’s course and therapeutic response.

**Figure 3:**
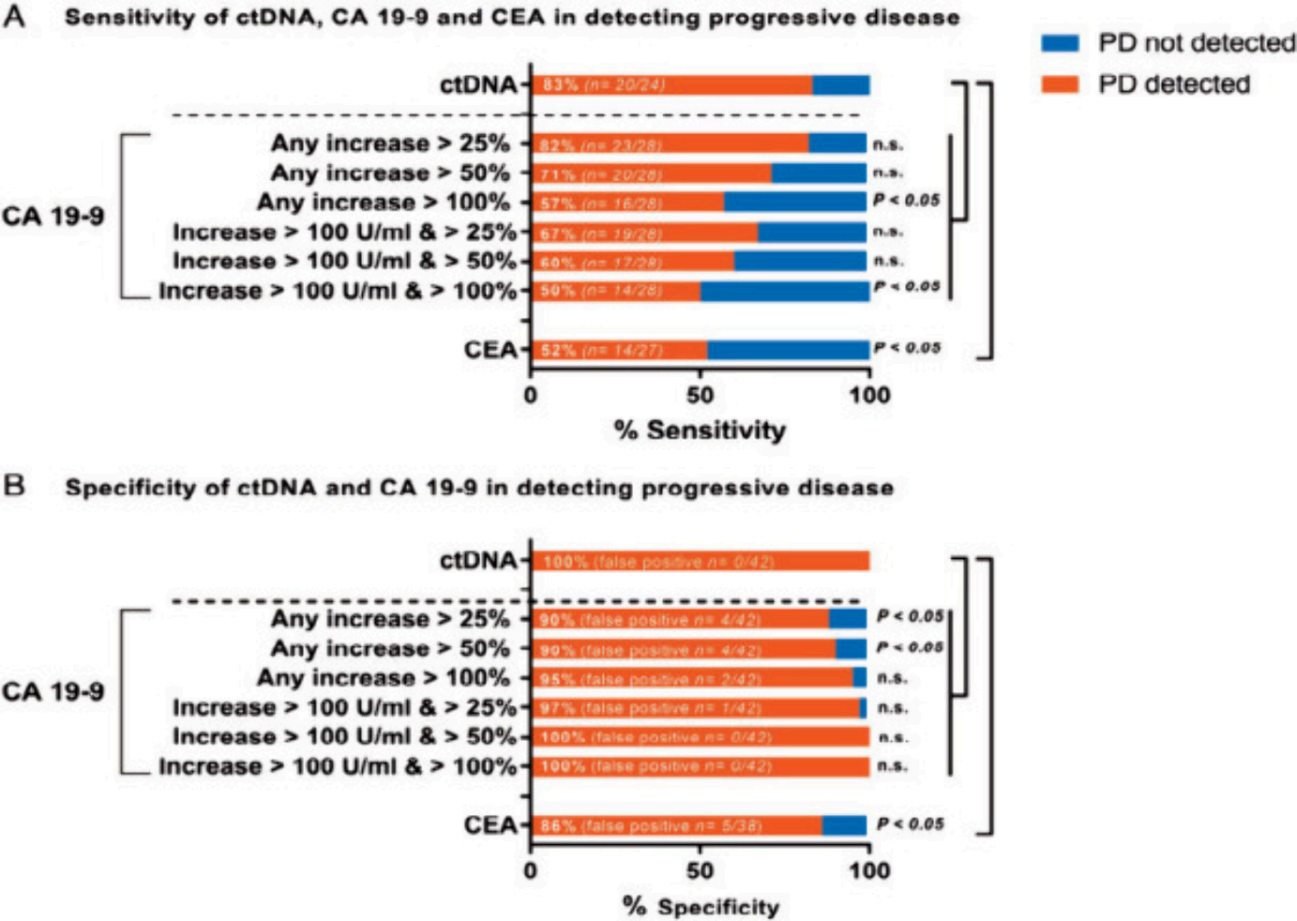
Sensitivity and specificity of mutKRAS ctDNA, CA 19–9 and CEA in detecting progressive disease. (A and B) For mutKRAS ctDNA any increase from baseline during chemotherapy was considered meaningful; for CEA any increase >1 ng/mL from baseline was considered meaningful; for CA 19–9 different cutoff values were tested as indicated. Chi-square test was used to test for statistical significance between mutKRAS ctDNA and CEA or the different cutoff values for CA 19–9, respectively. The presence of mutKRAS ctDNA, as well as higher levels of CA 19–9, CEA and CYFRA 21–1 before initiation of the first-line chemotherapy, was significantly correlated to an adverse overall survival. Chi-square test was used to test for statistical significance between mutKRAS ctDNA and CEA or the different cutoff values for CA 19–9, respectively. Adapted with permission from ref (27) Copyright © 2018 THE AUTHORS. Published by Elsevier Ltd.

A study by Kruger et al. investigated the prognostic value, therapy monitoring of patients and early response prediction with advanced pancreatic cancer using serial KRAS ctDNA readings.^27^ The objective of the study is to ascertain whether mutant KRAS ctDNA measurement levels and kinetics can provide more precise data on a patient’s prognosis and response to therapy than current standard-of-care indicators like CA19–9 and CEA. Chemotherapy based on gemcitabine was the first choice of therapy for these patients.^27^ The quantities and dynamics of mutant KRAS ctDNA with a mutated KRAS gene, are compared to the currently accepted standard-of-care indicators CA19–9 and CEA. The purpose of this comparison is to assess the efficacy of mutated KRAS ctDNA as a potential biomarker for anticipating chemotherapy response and tracking therapy in patients with advanced PC.^27^ The study sheds important light on the potential utility of mutated KRAS ctDNA as a biomarker for assessing chemotherapy response and therapy monitoring in patients with advanced PC **(**Figure 3**)**.^27^

According to the study, mutant KRAS ctDNA may be a more accurate and focused biomarker for determining how chemotherapy would affect a patient’s advanced PC and for tracking their treatment.^28−30^ Kruger et al. identified KRAS mutations in PC3 using dPCR technology.^27^ They employed ctDNA KRAS detection and quantification by dPCR as a stand-in marker of tumor load in PC to forecast response and track treatment.

In another study by Hussung et al., “hot spot” KRAS mutations were tracked by serial ddPCR-based ctDNA testing on 25 patients with resectable pancreatic ductal adenocarcinoma (PDAC).^31^ Their post hoc study found a significant correlation between overall survival (OS) and shorter recurrence-free survival (RFS), adopting a stricter MAF criterion (15 copies/mL of plasma). Notably, a decline in OS was linked to an increase in mutant KRAS discovered using ctDNA within the first 6 months following resection. This shows that postoperative ctDNA monitoring could potentially exceed CA 19–9 as a useful technique for predicting PDAC recurrence and OS. The extensive panel of 11 KRAS mutations used in the study supports its conclusions even more.^31^

Preoperative and postoperative ctDNA testing was used to monitor 97 patients with resectable and borderline resectable PC in a study by Yamaguchi et al.^32^ Regardless of the ctDNA status prior to surgery, they discovered that a positive postoperative ctDNA status was linked to a significantly shorter recurrence-free survival (RFS) for KRAS mutations namely G12D, G12 V, and G12R. This difference was 6.9 months as opposed to 19.2 months for individuals whose postoperative ctDNA status was negative. Notably, patients with positive pre- and postoperative ctDNA testing had significantly shorter RFS than patients with negative pre- and postoperative ctDNA testing.^32^ This demonstrates the predictive significance of ctDNA in estimating RFS in patients with pancreatic cancer.

### 2.3 Nonsmall Cell Lung Cancer

With numerous annual diagnoses and fatalities, lung cancer is a common and deadly disease. The majority of instances of nonsmall cell lung cancer (NSCLC), or adenocarcinoma, make up half of all cases of lung cancer. Sarcomatoid carcinoma, Adenosquamous carcinoma and nonsmall cell neuroendocrine tumors are further subcategories of NSCLC. Nearly 2 million new cases of lung cancer were identified in 2012, making it the most prevalent cancer in the world.^33^ Surgery, chemotherapy, radiation, and immunotherapy are among current NSCLC treatments.

To comprehend the molecular heterogeneity and clinical consequences of NSCLC across time, more study is necessary.^34^

#### 2.3.1 Circulating Tumor DNA as a Biomarker for NSCLC

ctDNA in the blood is a valuable plasma biomarker for NSCLC and has multiple applications such as early detection, monitoring, and therapy prediction.^35^ One option for treating patients with NSCLC is to use targeted drugs, but determining which patients will respond to treatment, needs the development of biomarkers. Relevant biomarkers for NSCLC include EGFR, ALK, ROS-1, and PD-L1.^36^

##### 2.3.1.1 EGFR Biomarker

**Figure 4:**
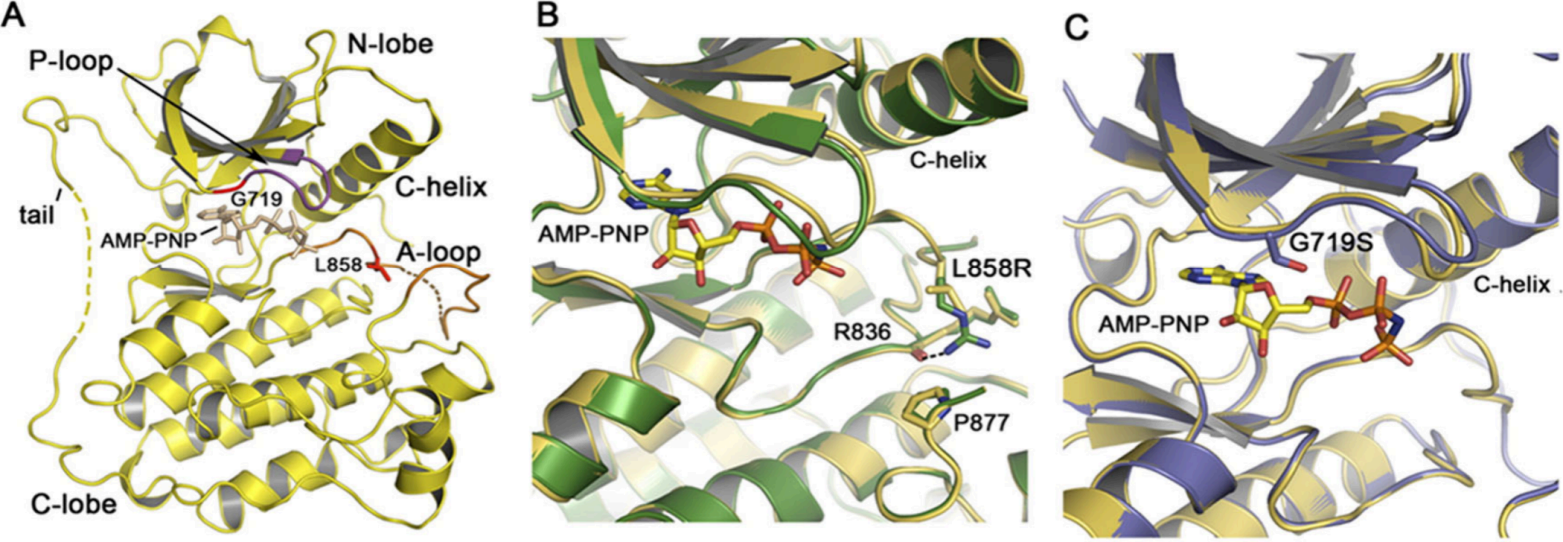
(A) Overview of the structure of the EGFR kinase. The structure of the wild-type kinase is shown in complex with the ATP analog AMP-PNP. The locations of the L858R and G719S mutations in the activation loop (A loop) and P loop, respectively, are indicated. (B) The structure of the active site region of the L858R mutant (green) superimposed on the wild-type kinase (yellow). (C) The structure of the active site region of the G719S mutant (blue) superimposed on the wild-type kinase (yellow). Adapted with permission from ref (40) Copyright © 2007 Elsevier Inc.

Mutational analysis of EGFR (also known as HER1) is one of the most widely investigated NSCLC biomarkers and one of the most often used in clinical practice.^35^ ctDNA provides a noninvasive alternative for mutational analysis, with good concordance between tissue and ctDNA for detecting EGFR mutations. EGFR mutations in NSCLC tissue can be found with high specificity using ctDNA assays, which makes it a reliable biomarker. Patients who test positive for EGFR mutations using ctDNA may benefit from EGFR tyrosine kinase inhibitor therapy (TKIs). Tissue analysis should be carried out, as a negative ctDNA result does not always imply that the matching tumor is mutation-negative.^37−39^ Deletion of Exon 19 (exon19del), which accounts for over 44% of EGFR mutations, insertion of exon 21 (L858R), which accounts for roughly 40% of EGFR mutations and G719S are the most common EGFR mutations in nonsmall cell lung cancer (NSCLC) **(**Figure 4**)**.^40^ Exon 20 insertions (ex20ins), which are present in roughly 10% of mutant EGFR NSCLC cases, are another less frequent mutation.^41,42^ Other rare EGFR mutations include S768I, G719X, and L861Q.^43^ All of these mutations are considered to as activating mutations because they enable EGFR to signal more frequently and consistently, which could result in the development and spread of cancer.

### 2.4 Lymphoma & Hodgkin Lymphoma

**Figure 5:**
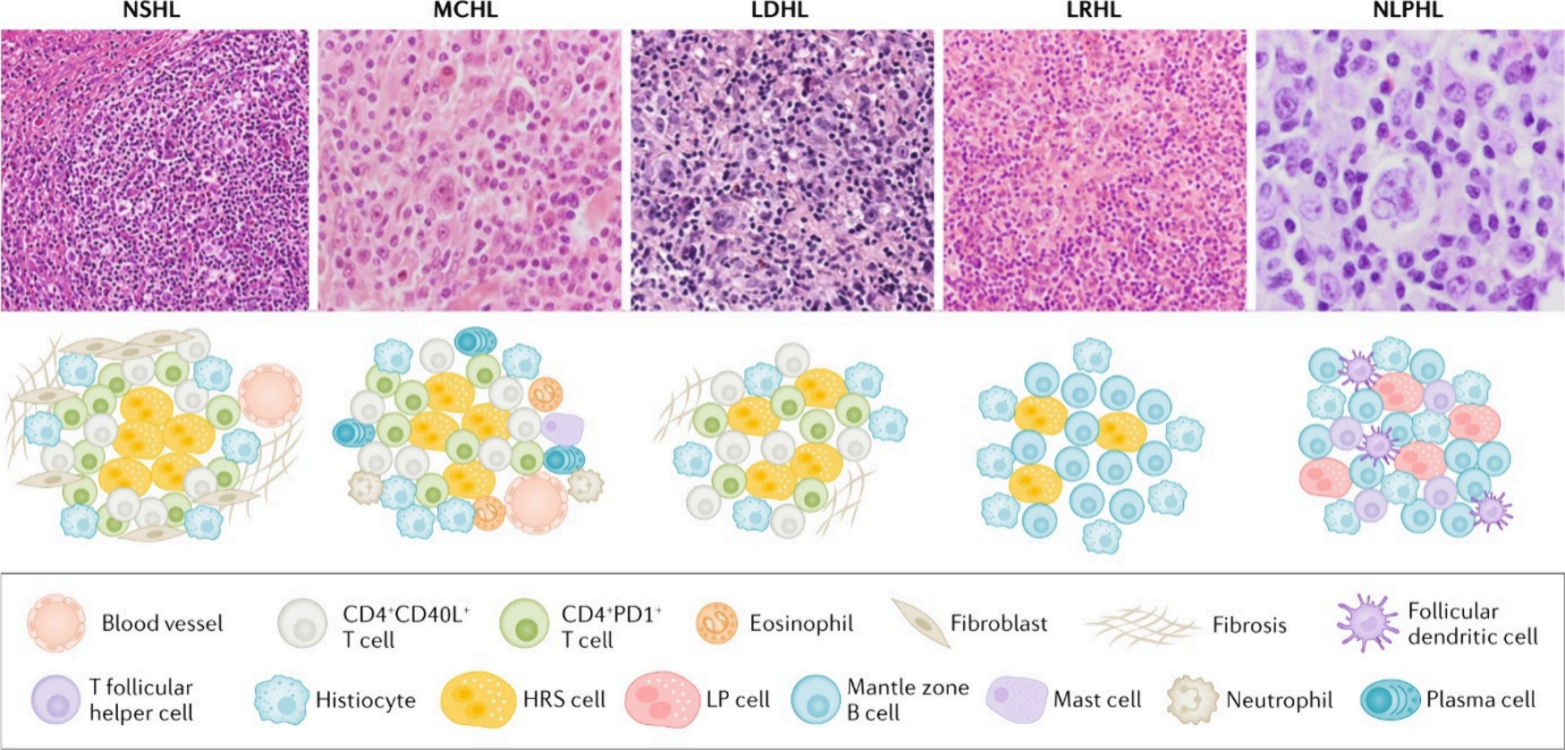
Histology images and corresponding drawings showing the cell types of TME of the four subtypes of Hodgkin lymphoma and the nodular lymphocyte-predominant Hodgkin lymphoma (NLPHL). In nodular sclerosis Hodgkin lymphoma (NSHL), the TME is specifically characterized by fibroblast-like cells and fibrosis. In mixed cellularity Hodgkin lymphoma (MCHL), the TME consists of a polymorphous reactive infiltrate with B cells and T cells, neutrophils, histiocytes, plasma cells and mast cells. In lymphocyte-depleted Hodgkin lymphoma (LDHL), the TME is usually composed of histiocytes and irregular fibrosis. In lymphocyte-rich Hodgkin lymphoma (LRHL), the TME is variable but usually consists of histiocytes and lymphocytes. The TME of NLPHL is similar to that of LRHL, although in NLPHL it is rich in follicular dendritic cells. Adapted with permission from ref (45) Copyright © 2020, Springer Nature Limited.

Hodgkin lymphoma (HL) is a relatively rare condition and has been classified into Classical Hodgkin lymphoma (cHL) and Nodular lymphocyte-predominant HL, which is detected more frequently, with an annual incidence of only a few new cases per 100,000 people in Western nations **(**Figure 5**)**. But among young individuals, it is one of the most prevalent cancer type.^44,45^ Most HL patients (80–90%) are curable with standard chemoradiotherapy.^46,47^ However, some individuals have a poor prognosis, particularly those who relapse after receiving first-line of therapy.^48^ Several lymphoma types have been associated with mutations in the genes exportin-1 (XPO1), EZH2, MYD88, BRAF, and RHOA.^49^

Hodgkin and Reed-Sternberg (HRS) cells, make up a small portion of the cellular infiltrate in Hodgkin lymphoma (HL) tumors. In HL tumors, the frequency of HRS cells ranges from 0.1 to 10,^50^ with the majority occurring at about 1.^51,52^ The residual tumor is made up of immune cells that have infiltrated the tumor and created the distinctively inflammatory milieu of HL tumors. Accurate diagnosis and pathobiology of Hodgkin lymphoma are largely dependent on the distinct cellular milieu, which is known to be a neoplasm produced from B cells.^53^ About half of instances of Hodgkin lymphoma cells show rearrangements of the BCL6 gene among other chromosomal abnormalities.^54−56^ Although HRS cells are produced from germinal center B cells, they do not display the CD19 and CD20 surface antigens, instead express the surface antigens CD30 and CD15 that are distinctive and are used for the diagnosis of cHL, (immunohistochemical stains- CD30 and CD15) as well as frequently utilized as markers.^57,58^ Cervical, mediastinal, supraclavicular, and axillary are the nodal locations that are frequently involved; however, there is some variance in site preference across distinct subtypes.^59^

#### 2.4.1 Circulating Tumor DNA as a Biomarker for Lymphoma & Hodgkin Lymphoma

ctDNA is a newly discovered biomarker for lymphoma, that even in the absence of radiographic disease, can detect little residual disease and offer further genotypic details, diagnostic clarification, and therapy prognostication.^60^ According to current research, ctDNA levels in cHL are correlated like in other aggressive lymphomas, with tumor volume on radiographic imaging.^61^ To evaluate the genetic basis of responsiveness and rejection of immunomodulatory therapy in clinical trials, ctDNA serves as an accessible and abundant source of tumor DNA for cHL mutation screening.^61^ The use of ctDNA in correlative studies and secondary end points for ongoing clinical trials is growing.

##### 2.4.1.1 XPO1 E571K Mutation as a Biomarker

Finding the mutation XPO1 E571K in plasma ctDNA should be investigated further in prospective research as it could be a potential biomarker for HL.^61^ Nevertheless, only 10–20% of individuals have XPO1 E571K, the only recurrent single mutation. Most HL lack a uniform biomarker for monitoring due to the lack of extremely widespread mutations.^61^ In a retrospective analysis, the XPO1 E571K mutation was found in ctDNA from patients with cHL harboring the reporter using NGS techniques and digital polymerase chain reaction (dPCR). It was discovered to be present in 24% of the patients. At the end of treatment, the presence of this mutation was associated with a shorter progression-free survival (PFS).^62^ As a result, quantifying ctDNA using the method of identifying tumor-specific mutations in cHL can be difficult or more challenging.^61^

According to a study, B-symptoms were associated with ctDNA levels in pediatric HL patients, and an increase in ctDNA levels following the first chemotherapy cycle was associated with a poorer prognosis.^63^ An additional investigation employing ctDNA NGS revealed genomic abnormalities in HL Reed-Sternberg (HRS) cells at the time of diagnosis. These imbalances were quickly corrected after therapy was started, indicating a potential function for ctDNA in early response monitoring.^64^ When it comes to cHL, ctDNA can monitor disease recurrence and could be a novel precision medicine biomarker or an early method of identifying patients who are chemorefractory. This was demonstrated in a study combining deep NGS-based ctDNA with PET imaging.^65^ To get more insight into the predictive and prognostic value of minimal residual disease (MRD) measurement utilizing ctDNA, more research is being conducted to monitor ctDNA in cHL pivotal trials.^60^

##### 2.4.1.2 Utilizing Circulating Tumor DNA as a Prognostic Baseline in Diffuse Large-B-Cell Lymphoma Patients

Since baseline ctDNA concentrations are associated with overall tumor burden in DLBCL, higher ctDNA levels are indicative of more tumors in the body. As a result, at the time of diagnosis, ctDNA can function as a prognostic marker, assisting in the prediction of a patient’s prognosis or chance of the disease progressing. Baseline ctDNA levels using NGS VDJ rearrangement sequencing were found to be correlated with radiographic staging of DLBCL patients, baseline lactate dehydrogenase levels and international prognostic index (IPI) scores in a study including 126 patients. This indicates that poorer prognostic variables were linked to increased ctDNA levels.^66^

A comparable ctDNA assay was used in another investigation, which discovered a correlation between ctDNA and the total metabolic tumor volume (TMTV) on the initial 18-Fluoro-deoxyglucose positron emission tomography scan. This indicates that a greater quantity of cancer was found in the body in correlation with higher ctDNA levels.^67^ Pretreatment ctDNA levels were substantially correlated with stage, IPI, and TMTVs in a large study of 267 Diffuse large-B-cell Lymphoma patients. Additionally, the study discovered a direct link between greater pretreatment ctDNA levels and a shorter diagnosis-to-treatment interval (DTI). Additionally, in multivariable Cox regression, ctDNA level was independent of DTI and IPI as a predictor of event-free survival (EFS). This indicates that, even after controlling for other variables that can have an impact on prognosis, higher ctDNA levels were linked to worse prognostic factors and worse outcomes.^68^ ctDNA can be used to distinguish between different clonal evolution models in converted DLBCL (see Tables 1 and 2).

**Table 1:** List of Various Categories of Lymphoma

Lymphoma Type	Abbreviation	Description
Diffuse Large B Cell Lymphoma	DLBCL	DLBCL is an aggressive lymphoma that develops from B cells and grows quickly.
Follicular Lymphoma	FL	B-cell lymphoma that grows slowly and frequently manifests as a painless swelling of lymph nodes.
Mantle Cell Lymphoma	MCL	The involvement of lymph nodes’ mantle zone is a characteristic of B-cell lymphoma.
Hodgkin’s Lymphoma	HL	Reed-Sternberg cells are an indicator of lymphatic system cancer.
Peripheral T Cell Lymphoma	PTCL	Diverse class of mature T-cell lymphomas that are frequently aggressive.
Primary Central Nervous System	PCSNL	Brain-related lymphoma predominantly impacts the central nervous system.

**Table 2:** List of Subcategories of Classical Hodgkin Lymphoma

Classical Hodgkin Lymphoma	
Subgroups	Description
Nodular Sclerosis HL	Characterized by fibrotic bands (nodular sclerosis) in the lymph nodes that are damaged.
Mixed Cellularity HL	Connected to a more sporadic pattern of involvement and a heterogeneous cell population.
Lymphocyte Depletion HL	Distinguished by an excess of Reed-Sternberg cells and a deficiency of lymphocytes.
Lymphocyte Rich HL	Has a high concentration of lymphocytes that are reactive, which results in a diffuse or nodular pattern.

### 2.5 Colorectal Cancer

In recent years, there has been a noticeable rise in the frequency of instances of colorectal cancer among those under 50 years of age. With 1,931,590 new cases reported globally in 2020, colorectal cancer accounted for 10% of all cancer cases and ranked third in terms of incidence. It also came in second place for the total number of deaths linked to cancer (935,173 deaths, or 9.4% of all cancer-related deaths). 52.3% of instances of colorectal cancer were in Asia, followed by North America (9.3%), Europe (26.9%), Latin America and the Caribbean (7%), and Africa (3.4%). Asia had the greatest incidence of colorectal cancer. Regional differences exist in colorectal cancer incidence and mortality rates, and these variations are strongly associated with the distribution of disease-related risk factors. There are differences in the characteristics of early onset colorectal cancer and colorectal cancer in older adults. Genetic (inherited gene mutations) and environmental (external variables that can raise the risk of cancer) factors work together to influence the development of colorectal cancer.^69^ It has a different frequency of mucinous histology (a particular type of tumor appearance), a different DNA methylation profile, a more distal location (tumor located further away from the beginning of the colon), and lower survival rates.^69^ The proliferation of mutations in specific signaling pathways ultimately leads to the onset and spread of colon cancer.^70^ These signaling pathways include TGF-beta (transforming growth factor-beta), P53, Wnt, and Epidermal growth factor receptor (EGFR).^71,72^ The methods that are now available for diagnosing disease progression are serum tumor markers, imaging (computed tomography [CT]), carbohydrate antigen 19–9 (CA19–9) and carcinoembryonic antigen (CEA).^73^

#### 2.5.1 Circulating Tumor DNA as a Biomarker for Colorectal Cancer

Because ctDNA provides information regarding the traits and behavior of CRC, it can be utilized to assess the dynamic properties of CRC.^74,75^ A patient’s ctDNA profile can be utilized to decide the best course of treatment, and it can also be used to track the effectiveness of therapy.^76,77^ It can also provide details regarding a patient’s chances of survival and prognosis.^78,79^ According to a meta-analysis of nonmetastatic cases, ctDNA may be a useful biomarker for the recurrence of postoperative tumors. This suggests that ctDNA may be able to predict whether a tumor will return following surgery.^80^ Changes in the amounts of mutated ctDNA in the blood can reveal whether the treatment is having an impact on the malignancy.^81,82^

One promising biomarker for CRC is hypermethylation of the neuropeptide Y gene (NPY) or meth-NPY. An effective method for measuring meth-NPY that can identify minute amounts of meth-ctDNA is Droplet digital polymerase chain reaction (ddPCR).^83,84^ Since hypermethylation of the NPY promoter region influences the transcription of the NPY gene, which is involved in cell invasion and proliferation, it has been proposed as a possible biomarker for colorectal cancer.^85,86^ Meth-NPY has been investigated in patients with metastatic colorectal cancer (mCRC) receiving first-line treatment as an early biomarker for treatment impact and surveillance.^87,88^ The majority of patients with mCRC have meth-NPY, which supports the adoption of this biomarker as a universal biomarker in CRC.^87^ Garlan et al., aimed to find an early marker of the impact of treatment on metastatic colorectal cancer.^89^ In 73 patients, the study examined mutant (KRAS, BRAF, or TP53) and methylation (WIF1 and NPY) ctDNA. The levels of ctDNA before the third treatment cycle and the baseline (before treatment) were compared by the researchers. When the levels of ctDNA were reduced to a minimal level of <0.1 ng/mL, the researchers observed a substantial change in both PFS and OS. Accordingly, PFS and OS were better for individuals whose ctDNA levels dropped to 0.1 ng/mL than for those whose levels remained higher.^89^ It may be possible to enhance patient outcomes and better manage metastatic colorectal cancer by using ctDNA as a marker of treatment effect.^87^

### 2.6 Liver Cancer

Hepatocellular carcinoma (HCC) and Cholangiocarcinoma are two different types of heterogeneous liver cancer. 85–90% of cases of primary liver cancer are HCC, making it the most prevalent kind. Its hallmark, malignant tumors of liver parenchymal cells, is a major cause of death, particularly in developing countries. Although it happens less frequently, cholangiocarcinoma, a tumor of the cells lining bile ducts, is nevertheless prominent in some nations. Liver cancer frequently arises as a result of liver cirrhosis and is linked to several etiologies, including chronic alcohol misuse, viral infections (hepatitis B and C), and metabolic syndrome. A tiny proportion of undifferentiated liver tumor cells known as liver cancer stem cells are essential for the development of cancer, metastasis, recurrence, and chemoresistance.^90−92^

#### 2.6.1 Gene Mutation as a Biomarker

The gene mutation is being investigated as a possible biomarker for the liver malignancy HCC. According to studies, HCC patients’ ctDNA frequently has mutations in genes including RAS, TERT, CTNNB1, TP53, AXIN1, and ARID1A.^93,94^ The majority of HCC patient’s tumor burden can be determined from somatic mutation sites ctDNA, which can also reveal details about the initial cancer biopsy.^93^

TP53 has the highest mutation rate with high HBV infection frequency in Chinese cohorts, while ARID1A has the maximum mutation rate in certain European cohorts. The frequency of these mutations may vary throughout cohorts.^95^ The identification of these mutations in ctDNA enables an entirely novel approach to tracking diseases and detecting cancer. It may also point in the direction of new treatment targets.^96^

#### 2.6.2 DNA Methylation as a Biomarker

Since DNA methylation has been linked to carcinoma pathogenesis and DNA regulation, and because it can appear in the early stages of tumor growth, it may be a biomarker for several types of human cancers. Utilizing the pervasiveness of DNA methylation produced for signal amplification, “Cancer Detector” has been utilized to predict multiple methylation statuses of many neighboring CpG islands on a single sequencing analysis. With the help of this technique, cancer can be early and sensitively detected through these methylation sites.^97^

Certain DNA alterations, such as CpG and 5hmC, can be utilized to identify methylation changes in ctDNA. It has been discovered that methylation sites on THY1 and DBX2 are very helpful as biomarkers for HCC detection and tracking. One method used to identify DNA methylation in circulating tumor DNA (ctDNA) in patients with early stage HCC is the Infinium Human Methylation 450 BeadChip.^98^

DNA methylation at the Gpbar1 (TGR5) has been found by Han et al. to be a potential biomarker for HCC patients with chronic hepatitis B (CHB).^99^ Serum MT1G and MT1M methylation was found in HCC patients far more frequently than in CHB patients or the healthy control group.^100^ Higher levels of INK4A promoter hyper-methylation may serve as a biomarker for patients with HCC since they develop early in the tumor’s course.^101^

#### 2.6.3 Protein Markers Combined with CtDNA

A novel liquid biopsy methodology named “CancerSEEK” was created for the early detection of tumors in a recent study by Cohen et al.^102^ This technique measures specific blood proteins in addition to analyzing mutations in ctDNA. Eight prevalent forms of cancer: lung, ovarian, stomach, pancreatic, esophageal, colorectal, and breast cancer were examined in this study. Test findings were positive in 70% of the 1005 cancer patients who underwent the procedure; the range of outcomes was 69% to 98%, depending on the kind of cancer. Comparing the test’s results to a control group of 812 healthy people, it was found that the specificity, or capacity to correctly identify healthy individuals, was greater than 99% for all the distinct types of tumors investigated.^102^ For liver cancer, the test’s sensitivity was about 98%. Significantly, the test’s sensitivity in identifying patients with stage I HCC, or early stage liver cancer, was nearly 100%.^102^

## 3 Electrochemical Biosensors for ctDNA Detection

**Figure 6:**
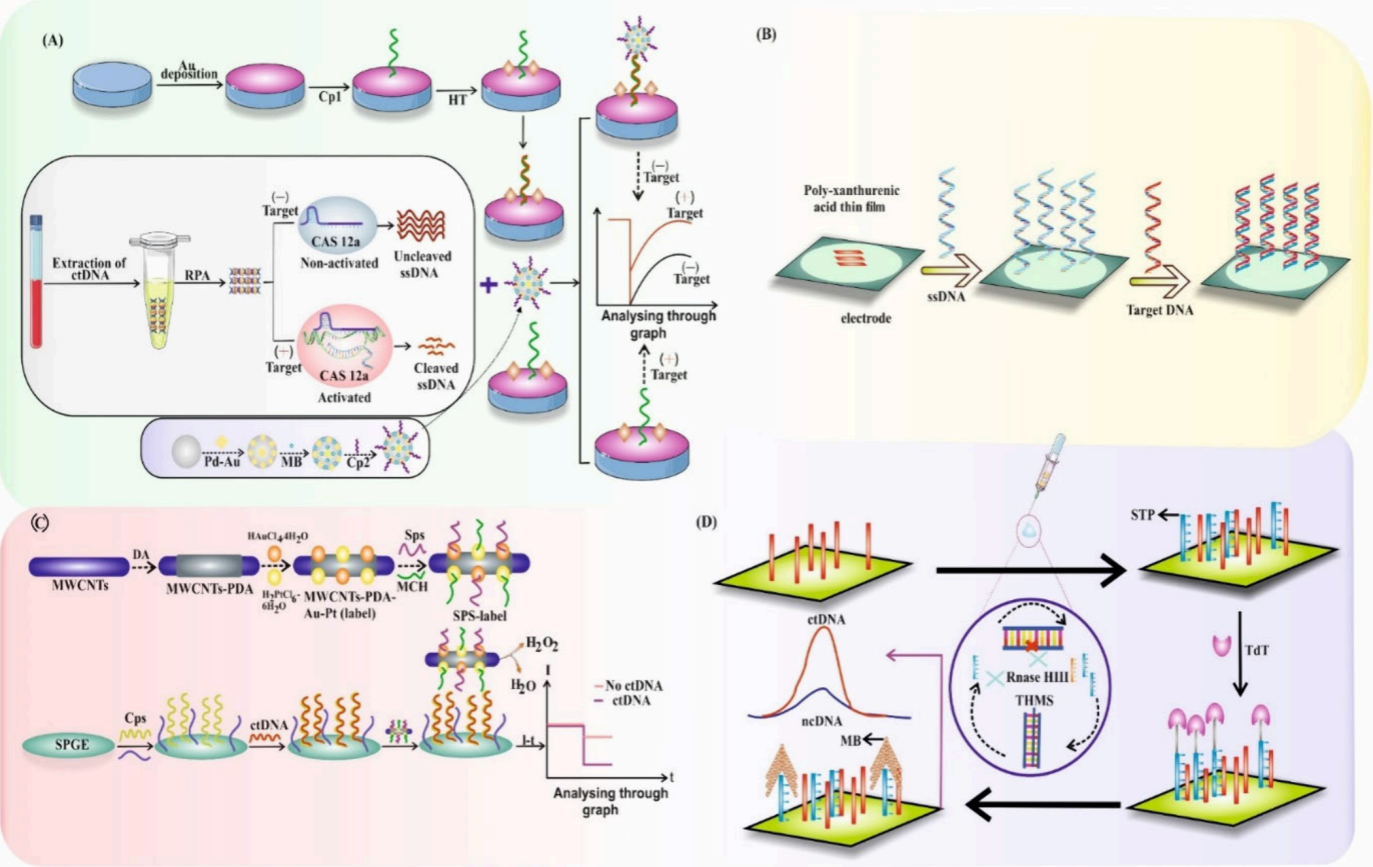
This diagram represents various electrochemical biosensors for the detection of circulating tumor DNA (ctDNA). Here, (A) Schematic diagram of ctDNA electrochemical biosensor based on CRISPR/Cas12a system (B) Polymer biosensor based on poly xanthurenic- acid functionalized MoS2 nanosheet (C) Schematic diagram of sandwich structure ctDNA electrochemical biosensor based on MWCNT-PDA–Au-Pt nanocomposite and Sps-label (D) Enzyme biosensor using THMS probe, TdT and RNase HII dual amplification. Parts of the figure have been drawn by using pictures from Servier Medical Art. Servier Medical Art by Servier is licensed under a Creative Commons Attribution 3.0 Unported License (https://creativecommons.org/licenses/by/3.0/.

Biosensor technology’s tiny size, low sample volume, quick detection time, high sensitivity, and accuracy have made it crucial for early cancer screening and tumor marker identification.^103^ The detection of ctDNA has drawn interest as a highly sensitive and specific diagnostic biomarker.^104^ Currently used ctDNA detection methods, such as label amplification depth sequencing, and digital PCR, have drawbacks in terms of test time, cost, portability, and false positive results.^105^ Electrochemical biosensors use an electrical signal to detect and analyze biological data. These biosensors function by attaching a specific recognition probe to the target ctDNA. When the recognition probe and the ctDNA bind, an electrical signal is produced that can be monitored and utilized for detection. Electrochemical biosensors measure the impedance of a range of frequencies or a particular frequency band to analyze biological data.^106^ The field-deployable capacity of electrochemical biosensors for ctDNA detection is greatly enhanced by their high sensitivity, fast response, portability, and specificity.^105^ The electrochemical characteristics of ctDNA are measured and analyzed using several techniques, including differential pulse voltammetry, square wave voltammetry, cyclic voltammetry, and electrochemical impedance spectroscopy **(**Figure 6). These techniques offer important insights into the diagnosis and management of cancer.^107^ Several nanomaterials have been utilized for the detection of ctDNA **(**Table 3**)**.

**Table 3:** List of Various Nanomaterials Used for the Detection of ctDNA

Methodology	Key findings	Reference
Graphene oxide covered gold nanoparticles on a glassy carbon electrode, fixed by the π–π contact between DNA bases.	Gastric cancer peripheral blood has been found to include ctDNA of the PIK3CA gene; the detection limit is low, at 1.0 × 10∧–20 mol/L, and there is potential for real-time detection in patient serum samples.	(108)
A poly xanthurenic acid film functionalized MoS2 nanosheet polymer biosensor.	Detection of the PIK3CA gene in gastric cancer peripheral blood; electropolymerized PXA on MoS2 electrode; alteration in self-signal following ssDNA hybridization with target DNA; 1.8 × 10∧–17 mol/L detection limit.	(109)
Triple-gene-negative breast cancer ctDNA detection via an electrochemical biosensor utilizing the nanocomposite MWCNT-PDA–Au-Pt	Triple-gene-negative breast cancer detection of ctDNA; amplification of nanocomposite; creation of sandwich structures; linear detection range from 1 × 10∧–15 mol/L to 1 × 10∧–8 mol/L; detection limit of 5 × 10∧–16 mol/L	(110)
TdT and RNase HII dual-enzyme cogroup amplification strategy-based circulating tumor DNA KRAS G12DM enzyme electrode biosensor	KRAS gene ctDNA detection in colorectal cancer patients; THMS as a molecular recognition probe; dual-enzyme cogroup amplification of TdT and RNase HII; extraordinarily accurate and sensitive detection; detection limit of aM.	(111)
Gold nanocrystals in the shape of sea urchins (U–Au) for target DNA-induced cyclic amplification.	Electrochemical response increased with ctDNA concentration from 0.1 fM to 1 × 10∧6 fM; detection limit of 0.033 fM; KRAS gene ctDNA detected in colorectal cancer; U–Au-modified multigraphene aerogel; cyclic amplification.	(112)
DNA nanostructure transition silicon nanowire array sensors on silicon-on-insulator (SOI).	PIK3CA E542 K ctDNA detection; SiNW array sensors; base complementary pairing and DNA bipedal walker; ultralow detection limit of 10 aM; ctDNA concentration detection range of 0.1 fM to 100 pM; excellent linearity.	(113)
To specifically recognize ctDNA, base complementary pairing, and DNA bipedal walking are used in DNA nanostructure transformation.	ctDNA detection from clinical samples; base complementary pairing and bipedal walking in DNA; DNA construction on electrode surfaces with CC, EIS, CV, and SWV characteristics; robust specificity with a detection limit of just 2.2 aM	(114)
Detection of ctDNA EGFR L858R using MB/Fe3O4@COF/PdAu nanocomposites and the CRISPR/Cas12a system.	Detection of CRISPR/Cas12a system, MB/Fe3O4@COF/PdAu nanocomposites, and ctDNA EGFR L858R; quantitatively detected based on change in current; linear range: 10 aM–100 pM; detection limit: 3.3 aM	(115)
ctDNA electrochemical biosensor utilizing AuPt/3D-GHC600 composite catalyst that has a low detection limit and high selectivity.	The ctDNA biosensor has a linear range of 10∧–8 M–10∧–17 M, a detection limit of 2.25 × 10∧–18 M, and high selectivity, repeatability, stability, and recovery.	(116)
	It is loaded with AuPt and synthesizes an AuPt/3D-GHC600 composite catalyst.	
HCR system included an electrochemical sensor.	ctDNA detection using the hybridization chain reaction (HCR) method, which has a 3 pM detection limit.	(117)
Electrochemical sensor with a ratiometric function.	ctDNA detection using electrochemical signal switching with a 25 aM detection limit.	(118)
Ti3C2MXene compound on ZnSe nanodisk.	Toehold-mediated strand displacement reaction as a novel research route for the electrochemical detection of ctDNA.	(119)

### 3.1 Detection Based on a Nucleic Acid Probe

**Figure 7:**
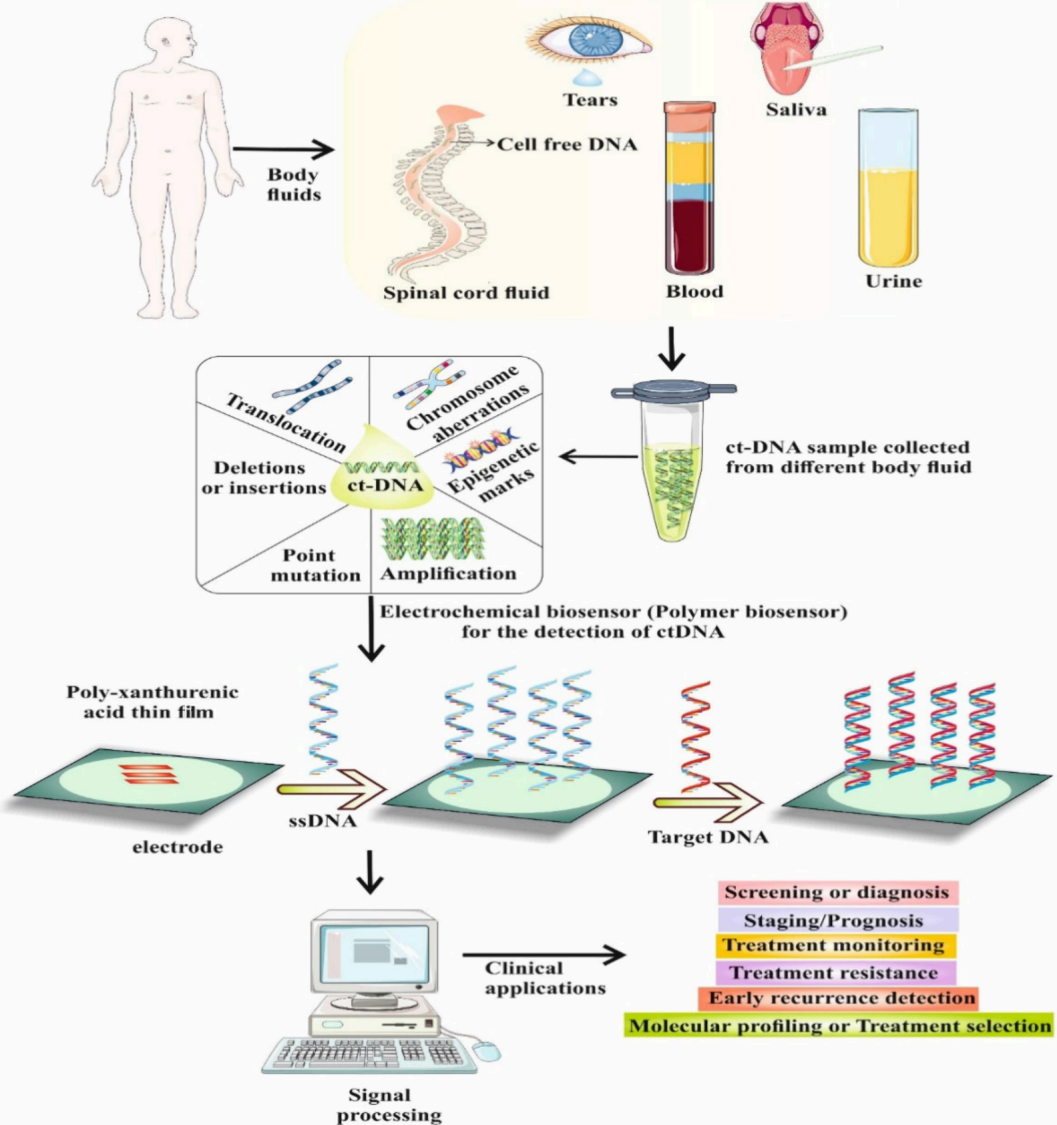
This schematic diagram represents collection of multiple body fluids, isolation of ct-DNA and analysis from collected body fluids, detection of specific cancer via electrochemical biosensing and screening, diagnosis, treatment monitoring, treatment resistance, recurrence detection and molecular profiling or treatment selection for specific cancer type. Parts of the figure have been drawn by using pictures from Servier Medical Art. Servier Medical Art by Servier is licensed under a Creative Commons Attribution 3.0 Unported License (https://creativecommons.org/licenses/by/3.0/.

In the field of biosensors, nucleic acid probe-based detection is a potent instrument that allows the very sensitive and accurate detection of specific DNA sequences.^107^ Biosensors for identifying nucleic acids are widely employed in many different disciplines, including environmental monitoring, microbiological detection, and clinical diagnostics. These probes, often comprising short sequences of single-stranded DNA or RNA, hybridize specifically to ctDNA fragments. Techniques like digital PCR and next-generation sequencing enhance sensitivity and specificity, allowing for the detection of low-abundance ctDNA. This approach aids in early cancer detection, monitoring treatment efficacy, and identifying minimal residual disease. The use of nucleic acid probes in liquid biopsies offers a promising, less invasive alternative to traditional tissue biopsies, enabling real-time insights into tumor dynamics **(**Figure 7**)**. In this regard three different probes are used: DNA probe, RNA probe and peptide nucleic acid (PNA probe) **(**Table 4**)**.

**Table 4:** List of Different Nucleic Acid Probes Used for Detection of ctDNA

Methodology	Key findings	Reference
Employing lead phosphate apoferritin and gold nanoparticle-fixed PNA probe, a dual biomarker detection platform is proposed.	Electrochemical detection utilizing SWV; measurement of ctDNA mutations and methylation; PNA probe attached on the sensor surface using covalent bond modification technology; Ultrahigh sensitivity and excellent selectivity; 1.0 × 10∧–14 mol/L detection limit.	(120)
DNA clutch probe combined with PNA probe-based ctDNA sensor to prevent ssDNA recombination.	DNA clutch probes for improved selectivity in preventing ssDNA recombination; ctDNA mutations seen in patients with melanoma and lung cancer; covalent bonding of PNA probe to a gold electrode for electrode potential measurement; and differential pulse voltammetry for measuring electrode potential changes.	(121)
ctDNA methylation and tumor-specific mutations: ultrasensitive detection utilizing PNA probe and LSPR.	Immunogold colloid used for enhanced secondary reaction; detection limit of 50 fM ctDNA with LOD of 4.3 nm LSPR peak offset in the range of 50–3200 fM; detection of E542 K and E545 K mutations and ctDNA methylation; coupled plasma model based on LSPR and gold nanoparticles.	(122)

#### 3.1.1 DNA Probe

DNA probes can identify target DNA molecules through certain hybridization interactions, they are frequently utilized in nucleic acid identification biosensors. To detect the ctDNA of the PIK3CA gene in the peripheral blood of gastric cancer patients, Rahman et al., developed an Au nanoparticles glass electrode and fixed the targeted DNA probe.^108^ Following the hybridization of the probe DNA with the target ctDNA and the formation of helical structure results in the detachment of the hybridized DNA molecule. This detachment leads to an increase in electrical current which can be detected using biosensors. This technique can be used to analyze ctDNA in serum samples from cancer patients in real-time. Genome hybridization reactions between target and probe DNA can be converted into electrical signals for examination in electrochemical biosensors by using DNA probes. A high-performance detection platform for ctDNA in blood was developed by Zhang et al., using polymer functionalized MoS2 nanosheets.^109^ The nanocomposite served as a substrate for the immobilization of DNA sequences specific for hybridizing with ctDNA. The DNA probe biosensor was found to be highly sensitive with a detection limit of 8 × 10^–17^ mol/L.^109^ A quick, precise, and economical assay for the identification of ctDNA EGFR L858R was developed by Liu et al., using the CRISPR/Cas12a system and PdAu/Fe3O4 nanostructure.^115^ The accurate identification of target ctDNA targets was possible by the role of the CRISPR/Cas12a system. The detection limit for this unique DNA probe was 3.3 aM.^115^

#### 3.1.2 RNA Probe

Short RNA segments that are compatible with a particular target DNA or RNA sequence are known as RNA probes. In molecular biology, it is employed to locate and recognize particular nucleic acid sequences. For visibility and detection, RNA probes might be marked with a fluorescent or radioactive tag. In situ hybridization and Northern blotting are two typical procedures that employ them. RNA probes are useful instruments in the field of genetic research and diagnosis.^107^

To detect ctDNA without the need for a label, Uygun et al. developed a biosensor that combines the inactivated Cas9 (dCas9) protein with a synthetic guide RNA (sgRNA) modified on a graphene oxide screen-printed electrode. Tumor-associated mutations in ctDNA were found by the biosensor using sequence-specific identification and electrochemical impedance spectroscopy (EIS) analysis. The impedance curve was lowered by covalently modifying the electrode with dCas9; however, the electron transfer resistance was raised by combining sgRNA modification with ctDNA. In 40 s, the biosensor modified with dCas9-sgRNA demonstrated a linear detection range of 2–20 nM for 120 bp ctDNA. With the lowest quantification limit (LOQ) of 1.92 nM and the lowest detection limit (LOD) of 0.65 nM, the biosensor showed good linearity.^123^

#### 3.1.3 PNA Probe

Because of its exceptional capacity to hybridize with DNA molecules, peptide nucleic acid (PNA) is employed as a probe in DNA sensors. DNA probes made of peptide nucleic acid (PNA) are frequently utilized. Because there is no electrostatic repulsion between PNA and DNA, PNA probes have a higher hybridization capacity than DNA–DNA interactions. The covalent bond modification method is used to fix PNA probes on the sensor surface. PNA probes use the Hoogsteen base-pairing principle, also known as complementary base-pairing, to create stable complexes with DNA.^107^ It makes it possible for the PNA probe to attach to and pick up target ctDNA molecules. In ctDNA electrochemical biosensors, the use of DNA probes—like PNA probes—offers great sensitivity, selectivity, and the possibility of real-time, portable ctDNA detection.^107^ Cai et al., introduced a dual biomarker detection platform based on lead phosphate apoferritin (LPA) and PNA probe-Au nanoparticles.^120^ This PNA probe could quantify ctDNA by detecting tumor-specific mutations and methylation of the PIK3CA gene. The detection limit of this probe was found to be 10^–15^ M.

### 3.2 Detection Based on an Antibody Probe

Specific binding by an antibody is the foundation for the detection of various biological molecules. The advantages of using an antibody probe for detection include a lower limit of detection and less nonspecific interference. Antibodies specific to ctDNA can be immobilized on the electrodes. The captured ctDNA from the body fluids could be then identified using an electrochemical technique. Microarray, sequencing and PCR are some of the techniques commonly used to analyze ctDNA methylation, however all these techniques require ctDNA pretreatment.^124,125^ On the other hand, monoclonal antibodies against 5-methylcytosinine could be covalently immobilized on the electrode, which can be hybridized with methylated ctDNA requiring no pretreatment of the sample. In a study 5-methylcytosinine and 5-hydroxymethylcytosine, the antibody was utilized to detect ctDNA.^126^ The implemented strategy enabled the sensitive and selective determination of the target methylated DNAs in less than 90 min, with high reproducibility for the simultaneous detection of the same or different cytosine epigenetic marks at the global level and in different loci of the same or different genes. Thus antibody based probes are a potential choice for ctDNA detection with ultra sensitivity.

## 4 Challenges and Prospects

Current ctDNA-based diagnostic techniques, particularly for tumors less than 10–15 mm in diameter, offer inadequate sensitivity for early cancer identification. A novel pan-cancer tumor marker ctDNA has important ramifications for monitoring patient prognosis, treatment response, and assessing progression. Another important goal is early cancer detection. Due to the low mutant allele fraction (MAF) and low tumor load, it is currently being investigated whether ctDNA can be used for early cancer detection.^127^ Although liquid biopsy enables the analysis of components of tumors present in bodily fluids like blood, it still has poor sensitivity.^128^ All ctDNA analysis techniques face difficulties due to the genetic diversity found in primary tumors and metastatic lesions, the evolution of tumors during treatment or surveillance, the presence of shared genetic mutations in precursor lesions, and the coexistence of germline mutations or clonal hematopoiesis.^129^ The amount of ctDNA from the tumor that is released into the bloodstream varies greatly according to the location, vascularity, cellular turnover, and stage of the tumor, among other things.^130,131^

Both biological challenges and technical restrictions are encountered while analyzing ctDNA in blood samples for cancer indicators.^132^ The contamination of genomic DNA (gDNA), which can affect the sensitivity of ctDNA analysis, is one well-known confounder.^133^ Although alternative techniques like capillary electrophoresis and quantitative polymerase chain reaction (qPCR) provide more accurate quantification, they may not always be able to identify enzyme inhibitors or gDNA contamination.^133^ The ability to discriminate between cfDNA fragments and gDNA is constrained by commonly used fluorometric methods for quantification.^134^ Alcaide et al. developed a multiplex single-well droplet digital PCR assay as a potential remedy for these problems.^133^ In pancreatic ductal adenocarcinoma (PDAC), Research has been done on ctDNA as a potential marker for cancer diagnosis and recurrence risk, but its clinical use is constrained by its poor yield. The low output of ctDNA may be caused by several factors, including low tumor cellularity, low DNA stability after release from tumor cells, and perplexing effects of DNA from nontumor cells, like white blood cells.^133^ Genetic changes and the heterogeneity of lymphoma subtypes may necessitate the creation of distinct ctDNA assays for various lymphoma types. Only CA 19–9, which can forecast treatment response and disease-free survival, has been integrated into the PDAC treatment paradigm. Although it can be increased in biliary diseases and is not secreted by tumors lacking the Lewis antigen, CA 19–9 lacks relative specificity.^135^

Standardized protocols for ctDNA detection can assist in developing consistency in sample processing, ctDNA extraction, and examination techniques, minimizing variability and improving result comparability throughout different studies and laboratories. Validation of ctDNA detection techniques is required to confirm its analytical performance parameters such as sensitivity, specificity, and precision.^127^ This validation indicates that the techniques can accurately identify tumor-related genomic changes in ctDNA. Researchers can increase the clinical value of ctDNA as a biomarker for different phases of tumor growth by developing strong validation criteria and standardized techniques and can overcome the current limitations of sensitivity and specificity particularly in samples with low ctDNA levels.^129^ In recent studies, the application of green biomaterials considerably advances cancer diagnostic technology by providing safer, more effective, and environmentally friendly solutions for early detection and follow-up of cancer treatment outcomes.^136^ Green biomaterials are great contrast agents for several imaging modalities due to their intrinsic optical, magnetic, and acoustic capabilities.

Combining ctDNA testing with different biomarkers or imaging methods offers great potential for cancer screening, prognosis, and therapy monitoring. Combining ctDNA analysis with other biomarkers, such as circulating tumor cells (CTCs), proteins, or microRNAs, may offer an understanding of tumor biology and dynamics.^2^ This comprehensive method improves cancer detection sensitivity and specificity while also predicting treatment response and disease progression. Furthermore, combining ctDNA analysis with modern imaging modalities such as PET, MRI, and CT scans provides a complementary approach to cancer detection and surveillance.^137^ Clinicians can improve their understanding of tumor load, heterogeneity, and responsiveness to therapy by connecting ctDNA mutations with imaging results. For high-risk individuals, ctDNA screening can lead to earlier interventions and improved prognosis. Additionally, for cancer survivors, regular ctDNA monitoring can promptly signal a recurrence, enabling swift treatment adjustments. Thus, ctDNA-based screening and monitoring are crucial for enhancing survival rates, personalizing treatment plans, and ultimately improving patient outcomes in oncology.

Circulating tumor DNA (ctDNA) detection via liquid biopsy represents significant advances in cancer diagnosis and treatment. However, it does raise serious concerns about genetic discrimination, such as the sensitive genetic information revealed to discriminate against people based on their genetic predisposition to cancer.^138^ To mitigate these risks, strict data protection measures are required. Ensuring the confidentiality and security of genetic data, implementing strong legal safeguards, and promoting ethical standards are critical for protecting individuals’ privacy and preventing discriminatory practices, thereby increasing trust in the use of liquid biopsy technology.

## 5 Conclusion

Cancer biomarker analysis is promising for enhancing molecular understanding of the disease, permitting more precise and prompt diagnosis and follow-up care. Researchers have taken an interest in medical -imaging, bioinformatics, and combining nanotechnology with biosensors which are highly sensitive to detect minute levels of cancer-specific molecules in physiological fluids. Integration with advanced bioinformatics techniques would allow for real-time analysis of complicated biological data, hence improving diagnostic accuracy. Furthermore, combining noninvasive imaging modalities would provide a comprehensive view, allowing clinicians to undertake targeted interventions more quickly.

Nowadays, ctDNA is recognized as an ideal tumor marker for being able to accurately representation of dynamic changes in early tumor screening, diagnosis, tumor molecular subtyping profiles, prognosis, recurrence tracking and effective drug selection. PCR and DNA sequencing are the two most widely used traditional clinical techniques for ctDNA detection; however, both techniques are not appropriate for point-of-care testing due to their high cost, complicated operations, limited sensitivity and numerous false positives. Tumor heterogeneity might not be effectively captured by ctDNA. ctDNA degrades due to which important genetic information can be lost during sample handling and processing. ctDNA represents a small fraction of total cfDNA which makes the detection challenging especially during early stage cancers. Furthermore, ctDNA assays’ sensitivity and specificity need to be improved to accurately identify and distinguish the tumor-derived DNA from noncancerous cfDNA.
